# Unraveling the Structure of Viral Replication Complexes at Super-Resolution

**DOI:** 10.3389/fpls.2013.00006

**Published:** 2013-01-31

**Authors:** Olga Linnik, Johannes Liesche, Jens Tilsner, Karl J. Oparka

**Affiliations:** ^1^Institute of Molecular Plant Sciences, University of EdinburghEdinburgh, UK; ^2^Faculty of Life Sciences, University of CopenhagenFrederiksberg C, Denmark; ^3^Biomedical Sciences Research Complex, University of St AndrewsFife, UK; ^4^Cell and Molecular Sciences, The James Hutton InstituteDundee, UK

**Keywords:** PVX, viral replication complex, 3D-SIM, super-resolution, TGB proteins, endoplasmic reticulum, Golgi

## Abstract

During infection, many RNA viruses produce characteristic inclusion bodies that contain both viral and host components. These structures were first described over a century ago and originally termed “X-bodies,” as their function was not immediately appreciated. Whilst some inclusion bodies may represent cytopathic by-products of viral protein over-accumulation, X-bodies have emerged as virus “factories,” quasi-organelles that coordinate diverse viral infection processes such as replication, protein expression, evasion of host defenses, virion assembly, and intercellular transport. Accordingly, they are now generally referred to as viral replication complexes (VRCs). We previously used confocal fluorescence microscopy to unravel the complex structure of X-bodies produced by Potato virus X (PVX). Here we used 3D-structured illumination (3D-SIM) super-resolution microscopy to map the PVX X-body at a finer scale. We identify a previously unrecognized membrane structure induced by the PVX “triple gene block” (TGB) proteins, providing new insights into the complex interplay between virus and host within the X-body.

## Introduction

### Viral replication complexes

In the process of host invasion, many plant viruses induce the formation of characteristic inclusion bodies that were initially termed “X-bodies” due to their unclear role (Goldstein, 1924). Variously referred to as amorphous inclusions, amorphous bodies, amoeboid bodies, vacuolate bodies, or viroplasms, such inclusion bodies were described in early studies by Goldstein (1926), Sheffield (1939, 1949). Inclusion bodies have been valuable in the diagnosis of plant virus diseases (Martelli and Russo, 1977; Edwardson and Christie, 1978), and many detailed studies of their structure were conducted using electron microscopy (Esau, 1967; Shalla and Shepard, 1972; Christie and Edwardson, 1977). Although the observation of inclusion bodies during infection provided some insight into their role, their detailed structure and function was a mystery until the arrival of molecular tools.

Plant viruses predominantly have positive sense, single-stranded RNA genomes ((+)ssRNA; Hull, 2002). (+)ssRNA viruses replicate on the cytoplasmic surfaces of modified host cell membranes, and many viral inclusion bodies have been revealed to be “virus factories,” i.e., replication sites (Miller and Krijnse-Locker, 2008; den Boon et al., 2010; Laliberté and Sanfaçon, 2010). Accordingly, these viral structures are now mostly referred to as viral replication complexes or VRCs (Asurmendi et al., 2004).

Viral RNA (vRNA)-dependent RNA polymerases (“replicases”) are usually active as oligomeric arrays (Lyle et al., 2002; Kopek et al., 2007; Spagnolo et al., 2010), and the host membranes they occupy serve as scaffolds to assemble these complexes (Nishikiori et al., 2006). However, the functions of VRCs are more complex than simply functioning to anchor replicase proteins to membranes. In addition to vRNA and proteins, they often incorporate host components including rearranged host membranes (Schaad et al., 1997; Carette et al., 2000; Dunoyer et al., 2002; Ritzenthaler et al., 2002; Zamyatnin et al., 2002; Turner et al., 2004) that form a sheltered environment for the viral genome (Miller and Krijnse-Locker, 2008; den Boon et al., 2010; Laliberté and Sanfaçon, 2010). Besides being the primary centers of viral replication, VRCs may also facilitate viral access to essential host resources such as ribosomes, enzymes, and nucleotides. In animal RNA viruses, viral packaging may be closely linked to viral egress via the secretory pathway and budding from the plasma membrane (den Boon et al., 2010). Similarly in plant viruses, VRCs could be sites of assembly of movement-competent ribonucleoprotein complexes (RNPs) for intercellular transport via plasmodesmata (Schoelz et al., 2011; Tilsner and Oparka, 2012). With such a complex variety of processes coordinated in close proximity within VRCs, a detailed knowledge of the spatial organization of host and viral factors is crucial to understanding the functions of VRCs. Renewed ultrastructural investigations, using electron tomography, have yielded high-resolution “maps” of the VRCs of Flock house virus (FHV) and SARS corona virus (Kopek et al., 2007; Knoops et al., 2008). However, similar studies are lacking for plant viruses. In the case of FHV, combination of tomographic and biochemical data enabled estimations of the numbers of replicase molecules and (−)RNA replication templates in the membrane invaginations that harbor the replication machinery (Kopek et al., 2007). However, electron microscopy is limited in its ability to localize specific macromolecules within VRCs. This is more easily done using fluorescence microscopy coupled to fluorescently labeled antibodies or fluorescent protein fusions.

Until recently, confocal laser scanning microscopy provided the highest resolution possible in fluorescence microscopy, with maximum resolutions of ∼200 nm in the focal plane (*x*-*y*) and ∼500 nm along the focal axis (*z*; Huang et al., 2009). Such ideal resolution is rarely achieved in heterogenous, living specimens, and for practical purposes confocal microscopy has approximately 50- to 100-fold lower resolution than electron microscopy, resulting in an inability to use confocal microscopy for structural mapping.

In recent years, various “super-resolution” microscopy (nanoscopy) approaches have been developed that overcome the diffraction barrier that limits conventional light microscopy, enabling fluorescence imaging at resolutions smaller than the wavelength of the emitted light (Huang et al., 2009; Schermelleh et al., 2010). Hence, these technologies are ideally suited to gain new insights into the structure-function relationships of VRCs (Horsington et al., 2012; Malkusch et al., 2012; Pereira et al., 2012). In practical terms, however, not all approaches are equally well suited to plants. In particular, the cell wall limits penetration of antibodies into plant cells. Therefore, the use of a genetically encoded fluorescent reporter fused with a protein of interest that is transcribed within the cell provides a better approach for intracellular studies. Additionally, the autofluorescence background created by chloroplasts and cell walls is particularly problematic for approaches that require single-molecule imaging such as photoactivation localization microscopy (PALM) and stochastic optical reconstruction microscopy (STORM; Tilsner and Flors, unpublished).

By contrast, three-dimensional structured illumination microscopy (3D-SIM) is a widefield imaging approach that is amenable to most specimens suitable for confocal microscopy. In 3D-SIM, a diffraction grating is superimposed upon the sample, and rotated during image acquisition. Sub-diffraction information is contained in the shifting diffraction patterns, and can be extracted by mathematical transformation, permitting image deconvolution with a resolution of ∼100 nm in *x*-*y* and 200 nm in *z* (Gustafsson et al., 2008; Huang et al., 2009). This constitutes an approximate two-fold increase in resolution over confocal microscopy, but in practical terms provides a significant increase in biological detail (Fitzgibbon et al., 2010; Phillips et al., 2012). We have previously used 3D-SIM to obtain super-resolution images of phloem sieve elements, including the localization of a viral movement protein to plasmodesmata (Fitzgibbon et al., 2010). To make the phloem accessible to 3D-SIM, we partially digested cell wall material and separated the cells of the tissue. Here, we employed 3D-SIM to analyze the X-body of a model virus, Potato virus X (PVX), and to demonstrate the suitability of the technique to imaging three-dimensional structures in leaf epidermal cells. This approach also should be suitable to a multitude of plant cell biology studies, including those conducted in the absence of virus infection.

### The Potato virus X-body

Potato virus X is a (+)ssRNA virus important for agriculture (Adams et al., 2004). It serves as a model virus for analysis of RNA silencing and virus movement, as a vector for protein overexpression and knockdown and as a virus-induced gene silencing model (Batten et al., 2003; Verchot-Lubicz et al., 2007). The mechanically transmitted PVX virions are flexuous filaments with a length of about 470–580 nm and are composed of the 6.4 kb vRNA and ∼1300 subunits of coat protein (CP; Atabekov et al., 2007).

**Figure 1:**
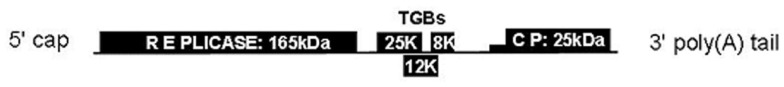
**Organization of the PVX genome (not to scale)**. TGB, triple gene block; CP, coat protein.

The PVX genome contains five open reading frames (ORFs) encoding five viral proteins (Batten et al., 2003): the 165 kDa replicase, which is the only viral protein required for replication (Doronin and Hemenway, 1996; Plante et al., 2000), a “triple gene block (TGB)” of three overlapping ORFs encoding the 25 kDa (TGB1), 12 kDa (TGB2), and 8 kDa (TGB3) movement proteins (MPs) responsible for cell-to-cell transport (Verchot-Lubicz et al., 2010; Solovyev et al., 2012 in this Research Topic), and the 25 kDa CP (Figure 1). All three TGBs and CP are needed for virus movement (Verchot-Lubicz et al., 2010) and CP is found in plasmodesmata and translocated between cells, indicating that it is a part of a movement-competent ribonucleoprotein complex (Oparka et al., 1996; Santa Cruz et al., 1998; Lough et al., 2000).

TGB1 is an RNA helicase that also functions as a translational activator (Atabekov et al., 2000; Rodionova et al., 2003) and silencing suppressor (Voinnet et al., 2000). TGB1 has been shown to be essential for forming the PVX X-body, and for recruiting actin filaments and host endomembranes [endoplasmic reticulum (ER) and Golgi] to this structure. TGB1 also recruits the two other viral MPs, TGB2, and TGB3 to the X-body (Tilsner et al., 2012). In contrast to TGB1, TGB2, and TGB3 are transmembrane proteins localized in the ER (Krishnamurthy et al., 2003; Ju et al., 2005). TGB2 induces the formation of ER-derived motile granules that also contain TGB3 (Ju et al., 2005, 2007; Samuels et al., 2007). The granules are associated with ribosomes, replicase, and virions (Ju et al., 2005; Bamunusinghe et al., 2009). As PVX replicates in association with the ER (Doronin and Hemenway, 1996), these granules may be replication sites.

Cells with mature PVX infections contain a perinuclear X-body. PVX X-bodies appear from about 1–2 days post-infection. They generally are circular or egg-shaped. The number and size of X-bodies per infected cell differs, but older infections typically contain only one. The X-body can be larger than the nucleus, ∼10–15 μm across, and is a complex amalgamation of host membranes including small vacuoles (Shalla and Shepard, 1972; Allison and Shalla, 1974; Santa Cruz et al., 1998; Tilsner et al., 2012). It also contains so-called “laminate inclusions” that are characteristic of PVX infection. In EM images, these inclusions consist of beaded or smooth sheets roughly 3 nm thick, firmly packed in several layers (Kozar and Sheludko, 1969; Stols et al., 1970; Shalla and Shepard, 1972; Allison and Shalla, 1974). Antibodies against TGB1 decorate the beaded sheets (Davies et al., 1993; Santa Cruz et al., 1998), and C-terminal fusions of fluorescent proteins (FPs) to TGB1 produce aggregates that morphologically resemble them (Tilsner et al., 2009, 2012). Thus, the inclusions contain large amounts of TGB1, but it is not clear if they consist entirely of the TGB1 protein. It was proposed that the beaded sheets could be active sites of viral protein synthesis (Kozar and Sheludko, 1969; Shalla and Shepard, 1972). The smooth sheets had virus particles between the layers of the sheets (Shalla and Shepard, 1972), whereas the beaded sheets did not (Stols et al., 1970; Shalla and Shepard, 1972). Whilst the beaded sheets superficially resemble ribosome-studded ER membranes, no lipids were found to be present in them, but treatment with potassium permanganate destroyed them, indicating that they are proteinaceous. The beads, found on both surfaces of the sheets, are too small to be ribosomes (Shalla and Shepard, 1972). Surprisingly, more recent work on TGB1 does not refer to these early data on TGB1 beaded sheets. Fluorescent fusions of TGB2 and TGB3 also localized to the X-body (Samuels et al., 2007; Tilsner et al., 2012). Lastly, encapsidated PVX virions surround the X-body and when the CP is fused to GFP, virions appear as fluorescent cages around the inclusions (Oparka et al., 1996; Santa Cruz et al., 1998; Tilsner et al., 2012).

Recently, we undertook a detailed structural and functional analysis of the PVX X-body and its biogenesis (Tilsner et al., 2012). The X-body is formed by gradual accumulation of the ER-derived, TGB2/3-containing granules around the TGB1 beaded sheets. Non-encapsidated vRNA, visualized with a fluorescent reporter construct *in vivo*, localizes to whorls that tightly encircle the TGB1 inclusions. The presence of “naked” RNA inside the X-body, and encapsidated virions at its periphery, along with the association of TGB2/3 granules with replicase, strongly suggested that the X-body is indeed a replication site, i.e., a VRC. In the absence of TGB1, no X-body is formed. Without an X-body, PVX still accumulates, but fewer virion aggregates are observed, indicating that the X-body may play a role in efficient virus encapsidation (Tilsner et al., 2012). In uninfected cells, ectopically expressed TGB1 can recruit TGB2 and TGB3 into a “pseudo-VRC,” which has a similar structure to the X-body.

In order to analyze the reorganized membrane structures of the PVX X-body at higher resolution, we turned to 3D-SIM microscopy. Here, we present results utilizing this technology to reveal new details of membrane organization within the PVX VRC and we demonstrate the applicability of 3D-SIM to general studies of plant subcellular structures.

## Materials and Methods

### Fluorescent reporter and virus constructs

Bombardment vectors for expression of TGB1-mCherry, GFP-TGB2, and TGB3-GFP, and binary vectors for agroinfiltration of TGB1-TagRFP, GFP-TGB2, TGB3-GFP, and unfused TGB2 and TGB3, as well as a binary vector for expression of a complete PVX genome with an endogenous TGB1-mCherry fusion were previously described (Ju et al., 2005; Tilsner et al., 2009, 2012). PVX.GFP-CP and PVX.mCherry-CP constructs were previously described (Santa Cruz et al., 1996; Tilsner et al., 2009). In some cases, a 35S promoter-driven PVX.GFP-CP bombardment construct (Christophe Lacomme, unpublished) was used for infections. A transgenic *Nicotiana* *benthamiana* line expressing ER-GFP (Haseloff et al., 1997), and a transgenic *Nicotiana tabacum* line expressing Golgi (ST)-GFP (Boevink et al., 1998), were described previously.

### Expression in plants

Infectious PVX RNA was obtained by T7 *in vitro* transcription from plasmid constructs containing PVX.GFP-CP and PVX.mCherry-CP modified cDNA copies, as described in Santa Cruz et al. (1996). Combinations of agrobacteria carrying binary expression constructs were infiltrated into *N. benthamiana* leaves at an OD_600_ of 0.15 or 0.25 each, as described previously (Tilsner et al., 2012). Microprojectile bombardments were carried out with a custom built gene gun according to the description in Gaba and Gal-On (2006).

### Imaging and image processing

Confocal microscopy was performed as described in Tilsner et al. (2009, 2012). For super-resolution imaging, lower epidermal peels were prepared using a pair of fine forceps to peel carefully but quickly an epidermal peel from the lower epidermis of *N. benthamiana* or *N. tabacum* plants. Along the length of the peels, thickness varied from a few cells to a single cell layer. Immediately after peeling, the epidermal peels were fixed by floating them in a fixative solution for 30–45 min at room temperature (for details see Fitzgibbon et al., 2010). The epidermal peels were assembled on a cover slip, not on a glass slide, in order to have the peel as close as possible to the cover slip. Finally, the peels were mounted in Citifluor AF1 antifade medium (Agar Scientific), pressing gently to remove residual Citifluor from under the cover slip. The samples were sealed with nail varnish, and viewed through a cover slip for 3D-SIM imaging with an OMX version 2 microscope (Applied Precision) as described in (Fitzgibbon et al., 2010). GFP was excited at 488 nm and TagRFP and mCherry were excited at 594 nm. Image processing was done as described in Fitzgibbon et al. (2010). Figures were assembled with Adobe Photoshop and ImageJ software. TGB2 and TGB3 membrane hoops and Golgi dimensions were measured using softWoRx (Applied Precision) software. Mean outer and inner diameters of the membrane hoops were compared by one-way ANOVA followed by Least Significant Difference and Duncan’s Multiple Range Tests using SPSS software (IBM).

## Results

### Fibrillar virion bundles surround the X-body

**Figure 2:**
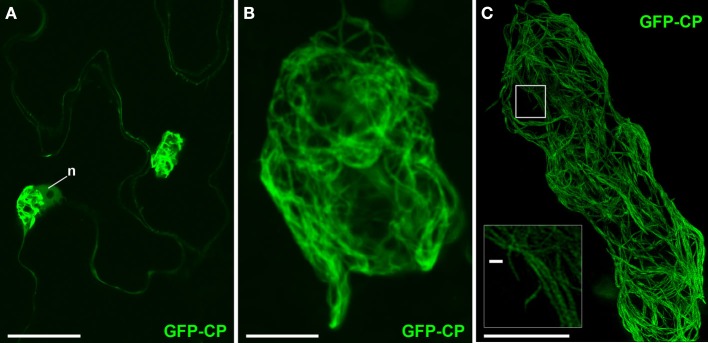
**PVX virion “cages” encasing the X-body**. **(A)** Live-cell confocal overview of PVX.GFP-CP-infected cells with two perinuclear (n: nucleus) X-bodies. **(B)** Higher magnification confocal image of a virion cage surrounding the X-body from a fixed sample. **(C)** High-resolution 3D-SIM image. The insert shows an enlargement of the area in the rectangle in which individual virion filaments are resolved to <100 nm diameter. Bars **(A)**: 50 μm; **(B,C)**: 10 μm; [insert in **(C)**]: 500 nm.

“Overcoat” PVX, in which viral CP is fused with a fluorescent protein via a 2A peptide linker, produces fluorescent virions in which a significant proportion (∼80%) of the virus coat is fluorescently labeled (Santa Cruz et al., 1996). The 2A peptide causes partial release of incomplete polypeptide without termination of translation, resulting in the production of both fluorescent protein-fused and unfused CP, thus enabling encapsidation. The fluorescent virions are found in fibrillar “cages” surrounding the X-body (Figure 2; Santa Cruz et al., 1998; Tilsner et al., 2012). In confocal images (Figures 2A,B), we observed large bundles of virus filaments but were unable to resolve the fine structure of the virion cages. Using 3D-SIM, we were able to resolve a fine network of virus bundles, the smallest of which were about 100 nm in diameter (Figure 2C insert). The diameter of individual PVX particles is 13 nm (Atabekov et al., 2007), suggesting that some of the small bundles that we resolved contained no more than eight virus particles aligned side-by-side. In three dimensions (Movie S1 in Supplementary Materials), the viral cages formed a complex interconnected network of virions that surrounded host and viral structures at its center.

### Super-resolution imaging of TGB1 aggregates at the center of the X-body

**Figure 3:**
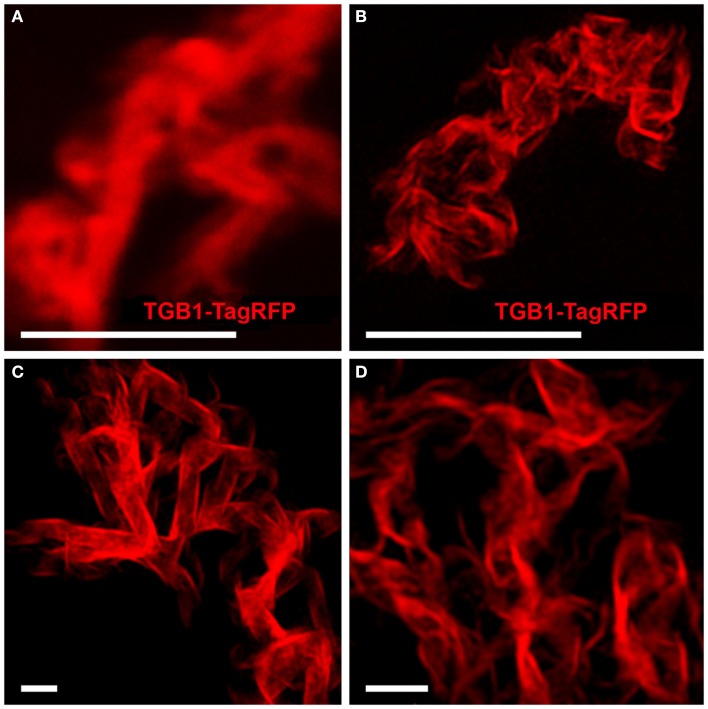
**TGB1 inclusions in the X-body**. **(A)** Aggregates of TGB1-TagRFP co-expressed with TGB2 and TGB3 (not shown) in pseudo-VRCs (Tilsner et al., 2012) from fixed, uninfected tissue, resolved by confocal microscopy. **(B–D)** 3D-SIM super-resolution images of the same material. **(A,B)** Shown at the same scale. Bars **(A,B)**: 5 μm; **(C,D)**: 1 μm.

TGB1 lies at the core of the X-body where it appears as walnut-shaped inclusions, each comprised of sickle-shaped aggregates (Figure 3; Tilsner et al., 2009, 2012). These correspond well to the circularly arranged TGB1 beaded sheets reported earlier from EM studies (Kozar and Sheludko, 1969; Stols et al., 1970; Shalla and Shepard, 1972; Davies et al., 1993; Santa Cruz et al., 1998). Using 3D-SIM we were able to resolve the fibrillar composition of the TGB1 aggregates, showing even more clearly their correspondence with the beaded sheets observed in EM (Figures 3B–D; Movie S2 in Supplementary Materials). Many of the TGB1 inclusions appeared to be arranged as flattened, undulating “ribbons” within the X-body (Figures 3C,D).

### Fine-scale architecture of the TGB2 and TGB3-induced membrane compartments within the X-body

**Figure 4:**
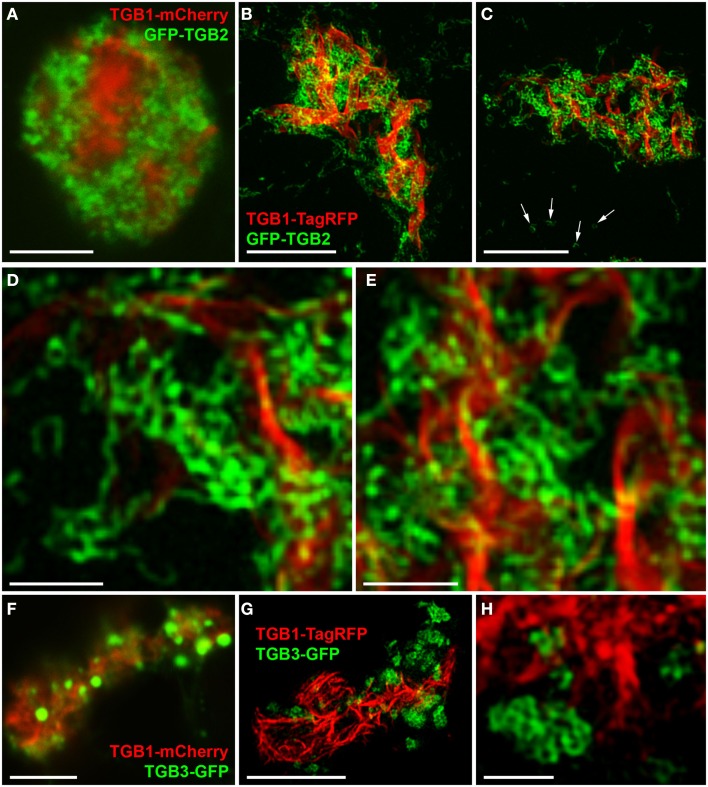
**TGB2- and TGB3-labeled membrane compartments in the X-body**. **(A)** Live-cell confocal image of co-bombarded TGB1-mCherry and GFP-TGB2 in PVX-infected cell. GFP-TGB2 signal is spread around the TGB1 aggregates. The granular appearance of the reorganized ER-derived membranes is not further resolved. **(B–E)** High-resolution 3D-SIM images of TGB1-TagRFP and GFP-TGB2 in a pseudo-VRC in an uninfected cell. GFP-TGB2-labeled membrane hoops form “chain mail”-like ribbons and dense arrays in the X-body, but are also observed on the cortical ER (arrows in **C)**. At higher magnification **(D,E)**, the hoop dimensions are apparent and the hoops can be seen winding around the TGB1 aggregates. **(F)** Live-cell confocal image of TGB1-mCherry and TGB3-GFP (co-bombarded into PVX-infected cells) show the occurrence of TGB3 granules or aggregates within the X-body. **(G,H)** In 3D-SIM images of TGB1-TagRFP and TGB3-GFP in a pseudo-VRC in an uninfected cell, the TGB3 structures are resolved as hoops similar in size to those labeled by TGB2 and concentrated in clusters or patches outside of the TGB1 inclusion. Bars **(A–C)**: 5 μm; **(D,E)**: 1 μm; **(F,G)**: 5 μm; **(H)**: 1 μm.

As previously reported (Tilsner et al., 2012), TGB2 and TGB3 surround TGB1 aggregates within the X-body. In confocal images GFP-TGB2 is broadly localized around the TGB1 inclusions, and this localization resembles the granulated morphology of the recruited ER membranes (Figure 4A; see also Tilsner et al., 2012). Unlike TGB2, the TGB3-GFP fluorescence is concentrated in isolated patches or clusters in the X-body (Figure 4F; see also Tilsner et al., 2012). The isolated patches of TGB3 probably correspond to the aggregated TGB2/3 granules of earlier infection stages (Bamunusinghe et al., 2009; Tilsner et al., 2012). Similar compartments were observed with ER-GFP and Golgi-GFP markers (Tilsner et al., 2012). We speculated previously that these compartments were comprised of densely stacked membrane sheets or tubules because both Golgi and TGB2/3 transmembrane markers labeled them completely, and not just their surface, as would be expected for vesicle- or vacuole-like membrane structures.

**Figure 5:**
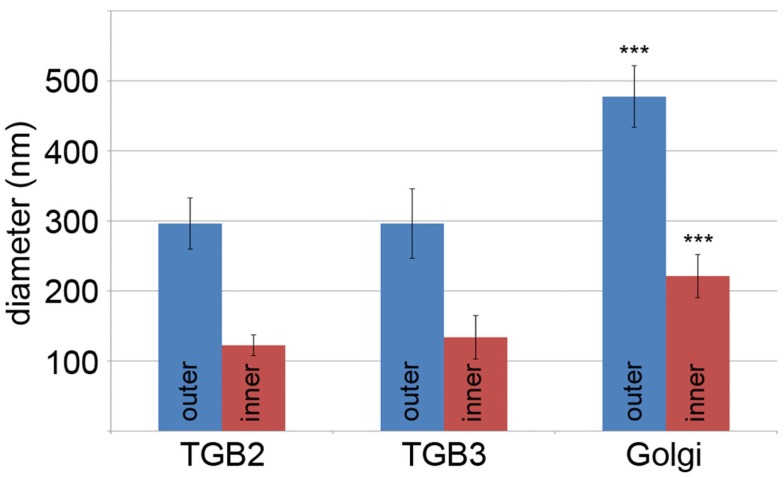
**Sizes of TGB2 and TGB3 membrane hoops and *trans*-Golgi rings**. Means with standard deviations are shown (TGB2: *n* = 8; TGB3: *n* = 21; Golgi: *n* = 17). Blue: outer diameter, red: inner diameter. TGB2 and TGB3 hoops outer and inner diameters, respectively, are not significantly different (*p* > 0.05), but both outer and inner diameter of Golgi rings are significantly different from both TGB2 and TGB3 (*p* < 0.001) (see Appendix for results of statistical analysis).

Using 3D-SIM, we now show that the “granules” produced by TGB2 and TGB3 are in fact fine membrane hoops of remodeled tubular ER. In confocal images, these structures had the characteristic granular appearance (Figures 4A,F) but under 3D-SIM they appeared as donut-shaped loops (Figures 4B–E,G,H) with an outer diameter of 296 ± 37 nm and an inner diameter of 123 ± 15 nm for TGB2 (*n* = 8; Figures 4D,E) or 296 ± 49 nm (outer) and 134 ± 31 nm (inner) for TGB3 (*n* = 21; Figure 4H), respectively. Outer and inner diameters of the TGB2 and TGB3 hoops were not significantly different (*p* > 0.05, Figure 5; see Appendix). The clear separation of the two membrane tubes on opposite sides of the hoops, with apparent diameters of ca. 80–90 nm, and separated by only ∼120–130 nm, indicates that a lateral resolution of less than 100 nm was achieved by 3D-SIM in these images. TGB2 hoops formed dense arrays resembling “chain mail” in the center of X-bodies, wrapped around the TGB1 inclusions (Figures 4B–E). TGB3 hoops were more concentrated in patches around the TGB1 inclusions (Figures 4G,H; Movie S3 in Supplementary Materials). We have previously shown that TGB2 is more dispersed over the ER within the X-body, but also co-localizes with TGB3, which is confined to granules or aggregates (Tilsner et al., 2012). These findings are corroborated here and the 3D-SIM data indicate that these different modified ER compartments are all comprised of dense arrays of membrane hoops containing either only TGB2 or both TGB2 and TGB3. We could detect these hoops also on the peripheral cortical ER (arrowed in Figure 4C), and these probably correspond to the previously reported TGB2-induced, ER-derived granules (Ju et al., 2005).

### Reorganization of endomembranes within the X-body

**Figure 6:**
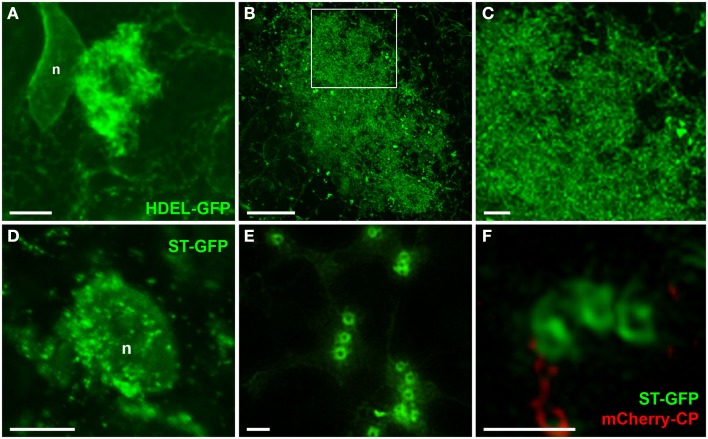
**Reorganized host endomembranes in the X-body**. **(A)** Confocal image of densely reticulated host ER within the PVX X-body and unmodified cortical ER network outside of the X-body in fixed tissue. ER is labeled with lumenally targeted HDEL-GFP (Haseloff et al., 1997). n: nucleus. **(B,C)**, Super-resolution images of remodeled ER in the X-body of cells infected with PVX. TGB1-mCherry (not shown).The area in the rectangle in **(B)** is enlarged in **(C)** and shows the dense arrays of ER membranes to consist of membrane hoops similar to those labeled by the TGB2 and TGB3 proteins. **(D)** Confocal image of Golgi stacks labeled with ST-GFP (Boevink et al., 1998) recruited to a nascent X-body of a PVX-infected, fixed cell. **(E,F)**, Super-resolution images of Golgi stacks in cells infected with PVX.mCherry-CP [not shown in **(E)**] resolve the trans-Golgi as a membrane circle with a larger diameter than the TGB2/3-containing ER hoops [note **(C,E)** have almost identical scales]. Bars **(A,B)**: 5 μm; **(C)**: 1 μm; **(D)**: 10 μm; **(E,F)**: 1 μm.

Changes in the morphology of host ER and Golgi membranes were also more clearly resolved by 3D-SIM than in previous confocal images (Figure 6, see also Tilsner et al., 2012). The individual tubules of the ER network are barely discernible in the X-body even though they are unaltered in the surrounding cytoplasm (Figure 6A). However, at high magnification, the diffuse membrane aggregations within the X-body consist of the same membrane hoops observed for TGB2 and TGB3 (Figures 6B,C), in agreement with the previously demonstrated ER-association of these proteins (Krishnamurthy et al., 2003; Ju et al., 2005).

3D-SIM also resolved individual Golgi bodies labeled with a sialyl transferase (ST)-GFP membrane marker (Boevink et al., 1998) and revealed a ring-shaped structure (Figures 6E,F). Such details of this organelle are not visible in conventional confocal microscopy (Figure 6D). ST-GFP is a trans-Golgi marker (Boevink et al., 1998) and the ring structure probably corresponds to the outer rim of *trans*-Golgi compartments viewed along the *trans-cis* axis (Staehelin and Kang, 2008). However the Golgi rings were clearly different from the ER-derived membrane hoops observed with TGB2 and TGB3. They had larger outer (478 ± 44 nm) and inner (221 ± 31 nm) diameters (Figure 5; *n* = 17; statistically significant at *p* < 0.001; see Appendix) which correspond well to EM observations (Staehelin and Kang, 2008), and did not form linked “chain mail” structures or large arrays. This is in agreement with previous biochemical and microscopical findings that there is no direct association between the TGB proteins and the Golgi apparatus (Ju et al., 2005; Bamunusinghe et al., 2009).

## Discussion

### Possible roles of remodeled endomembranes within the X-body

**Figure 7:**
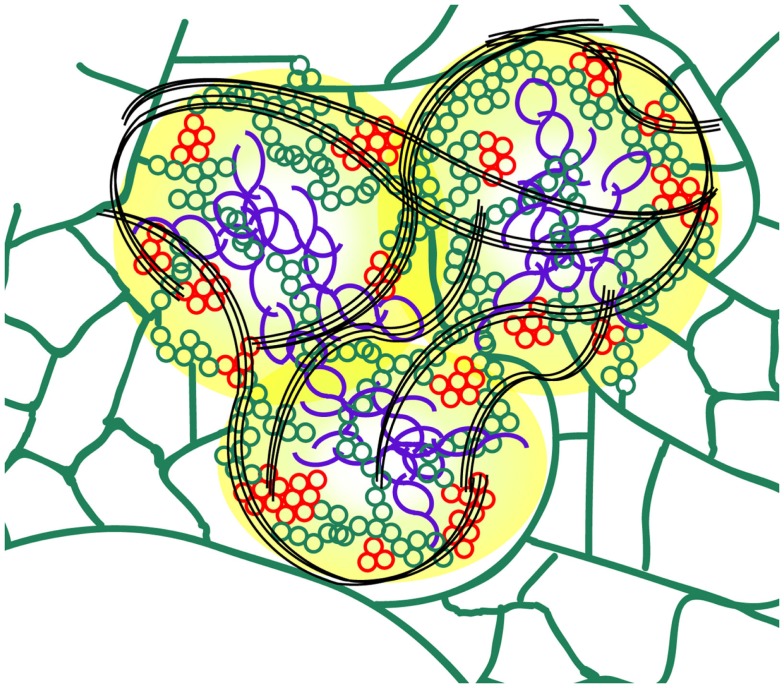
**Schematic model of the PVX X-body (not to scale)**. The TGB1 “beaded sheets” (purple) are localized in the center of the X-body. As shown previously, non-encapsidated vRNA (yellow) surrounds the TGB1 inclusions (Tilsner et al., 2009, 2012). Host ER (green) is remodeled into arrays of small membrane hoops by TGB2 which are wrapped around the TGB1 aggregates within the X-body. Some patches of these TGB2 loops also contain TGB3 (red) and may constitute the replication sites of the virus (Bamunusinghe et al., 2009). Bundles of encapsidated virions (black) accumulate at the periphery and form “cages” around the X-body sub-compartments.

In previous work we described the essential role of the TGB1 protein in generating the PVX X-body, and presented a model of the layered structure of this virus “factory” (Tilsner et al., 2012). The increased resolution provided by 3D-SIM enabled us to analyze in greater detail the TGB2 and TGB3 sub-compartments and the role of these proteins in organizing the X-body, and allowed us to update our previous model of the PVX “factory” (Figure 7). Our new data show that TGB2-labeled ER membranes consist of small hoops, which cluster within the X-body to form an extremely dense network. Since TGB2 and 3 are integral membrane proteins, the hoops are expected to be membrane structures. In previous studies (Boevink et al., 1996; Krishnamurthy et al., 2003; Mitra et al., 2003; Ju et al., 2005; Samuels et al., 2007; Wu et al., 2011) ER markers closely mirrored the locations of the TGB2 and 3 proteins, and we found that a lumenal ER marker also labeled small hoops in the X-body (Figures 6B,C). It can therefore be assumed that the TGB2/3 hoops remain within and are identical with the densely reticulated ER network within the X-body. The previously observed ER-derived TGB2/3 granules (Boevink et al., 1996; Ju et al., 2005) may in fact also be individual or small clusters of hoops branching out from the cortical ER (Figure 4C arrows). Within the resolution limits there is currently no evidence that the membrane tubules differ from those of the normal ER, however the “knitting” of the hoops is far more dense than in the unmodified cortical ER network, where three-way junctions are typically spaced a few μm apart, although reticulation of a similar density to the TGB2/3 hoops can also occur, for instance in meristematic cells (Boevink et al., 1998; Sparkes et al., 2009a,b). These observations suggest that TGB2 may remodel the ER by inducing a localized increase of network branching. The ability of the transmembrane TGB2/3 proteins of potexviruses to influence the structure of the ER requires further study. Recently, it was shown that a specific class of host proteins, the reticulons, is involved in the formation of VRCs by Brome mosaic virus replicating in yeast (Diaz et al., 2010; Diaz and Ahlquist, 2012). It will be interesting to see if this class of proteins is recruited to the X-body during PVX accumulation and whether reticulons, and other host proteins associated with ER-remodeling, operate in tandem with TGB2/3 type proteins.

Modification of host organelles and their redirection to, and involvement in, X-body organization is likely to be a vital event in the PVX infection process. One possible role of recruited host elements is to protect the virus from the host plant defense mechanisms by wrapping it in plant membranes and creating a unique isolated environment for the replicating virus in which it is more difficult for the plant to recognize and degrade the vRNA through the plant RNA silencing machinery. In addition, it is possible that the recruited host membranes enlarge the surface area for the replicating virus, making replication more efficient because of the production of increased concentrations of important viral components (Dunoyer et al., 2002; Schwartz et al., 2002, 2004; Sanfaçon, 2005; Laliberté and Sanfaçon, 2010). The dense arrays of membrane hoops observed by 3D-SIM are in agreement with this hypothesis. For a conclusive interpretation regarding the membrane surface utilized for replication, super-resolution localization of the PVX replicase will be required, and these methods are currently being developed in our lab. The organization of X-bodies is also thought to create a subcellular environment in which host resources required by the virus, e.g., translation factors, are readily available (Schwartz et al., 2002, 2004; Sanfaçon, 2005), and the reorganization of ER membranes may play a role in this. Detailed analyses of the interaction partners of the TGB2 and TGB3 proteins might corroborate this hypothesis for the PVX X-body. It is also possible that containment of viral replication in the X-body minimizes damage to the host cell (Sanfaçon, 2005). Lastly, endomembranes and cytoskeletal elements also provide the routes for viral cell-to-cell transport (Harries et al., 2009; Schoelz et al., 2011) and their reorganization by TGB proteins within the X-body probably reflects the movement-related activities of these proteins at earlier infection stages.

The accumulation of encapsidated virions on the cytoplasmic side of the X-body (Oparka et al., 1996; Santa Cruz et al., 1998; Tilsner et al., 2012; current study) suggests that CP synthesis and packaging of vRNA take place at the periphery of the X-body, whereas the location of the TGB proteins, in particular TGB1, may be influenced by both their targeting properties and their site of synthesis within the X-body (Tilsner and Oparka, 2012). To fully address these questions, the distribution of the subgenomic messenger RNAs required for translation of these proteins requires to be analyzed within the VRC. However, this is beyond the technical limits of current localization techniques. The distinct localization of PVX CP and TGB1 in the X-body and their putative production (and isolation) in separate sub-compartments is probably essential for PVX infection (Karpova et al., 2006). Because TGB1 destabilizes PVX virions *in vitro* (Rodionova et al., 2003), it needs to be sequestered away from those progeny virions destined for mechanical transmission to other host plants.

## Conclusion

3D-SIM “super-resolution” has enabled us to gain new insights into the structural organization of the replication “factory” of a model plant virus and develop new hypotheses about its functions. This highlights the value of super-resolution approaches for the analysis of other viruses, including those that infect animal cells. The study of viral inclusions is an area within cell biology that lends itself to the practical application of super-resolution microscopy, bringing its powers to bear on important biological questions. To obtain 3D-SIM images does not require complicated embedding and sectioning techniques but only mild fixation and the use of antifade reagents, ensuring a low degree of sample disruption. Imaging was conducted on intact epidermal cells in single- and even multi-cell layer epidermal peels, showing the versatility of 3D-SIM for complex biological specimens. Due to their greater photostability, we found GFP fusions better suited to 3D-SIM imaging than RFP constructs, but the rapid development of new FPs is likely to overcome such limitations in the near future, and others have successfully imaged RFP fusions with 3D-SIM (Horsington et al., 2012). The increased resolution gained, for example on Golgi bodies, demonstrates the utility of this approach outside virology. In the future, correlative super-resolution light and electron microscopy approaches (Fridman et al., 2012) should enable a complete mapping of virus “factories” and other complex cellular structures at near-molecular resolution.

## Conflict of Interest Statement

The authors declare that the research was conducted in the absence of any commercial or financial relationships that could be construed as a potential conflict of interest.

## Supplementary Material

The Supplementary Material for this article can be found online at http://www.frontiersin.org/Plant-Microbe_Interaction/10.3389/fpls.2013.00006/abstract

## Appendix

### Descriptives

**Table:**

	*N*	Mean (μm)	SD	SE	95% Confidence interval for mean		Minimum	Maximum
					Lower bound	Upper bound	
**OUTER DIAMETER**								
TGB2	8	0.2963	0.03662	0.01295	0.2656	0.3269	0.25	0.34
TGB3	21	0.2962	0.04944	0.01079	0.2737	0.3187	0.20	0.37
Golgi	16	0.4775	0.04405	0.01101	0.4540	0.5010	0.41	0.56
Total	45	0.3607	0.09843	0.01467	0.3311	0.3902	0.20	0.56
**INNER DIAMETER**								
TGB2	8	0.1225	0.01488	0.00526	0.1101	0.1349	0.11	0.14
TGB3	21	0.1338	0.03106	0.00678	0.1197	0.1479	0.10	0.20
Golgi	16	0.2213	0.03074	0.00769	0.2049	0.2376	0.17	0.28
Total	45	0.1629	0.05229	0.00780	0.1472	0.1786	0.10	0.28

### Test of homogeneity of variances

**Table:**

	Levene statistic	df1	df2	Sig.
Outer diameter	0.198	2	42	0.821
Inner diameter	1.420	2	42	0.253

### ANOVA

**Table:**

	Sum of squares	df	Mean square	*F*	Sig.
**OUTER DIAMETER**					
Between groups	0.339	2	0.169	81.444	0.000
Within groups	0.087	42	0.002		
Total	0.426	44			
**INNER DIAMETER**					
Between groups	0.085	2	0.043	51.153	0.000
Within groups	0.035	42	0.001		
Total	0.120	44			

### *Post hoc* tests, *p* < 0.001.

#### Multiple comparisons

**Table:**

Dependent Variable		(I) Factor	(J) Factor	Mean difference (I-J)	SE	Sig.	99.9% Confidence interval	
							Lower bound	Upper bound
Outer diameter	LSD	TGB2	TGB3	0.00006	0.01895	0.998	−0.0670	0.0671
			Golgi	−0.18125*	0.01975	0.000	−0.2511	−0.1114
		TGB3	TGB2	−0.00006	0.01895	0.998	−0.0671	0.0670
			Golgi	−0.18131*	0.01514	0.000	−0.2349	−0.1278
		Golgi	TGB2	0.18125*	0.01975	0.000	0.1114	0.2511
			TGB3	0.18131*	0.01514	0.000	0.1278	0.2349
Inner diameter	LSD	TGB2	TGB3	−0.01131	0.01200	0.351	−0.0538	0.0311
			Golgi	−0.09875*	0.01250	0.000	−0.1430	−0.0545
		TGB3	TGB2	0.01131	0.01200	0.351	−0.0311	0.0538
			Golgi	−0.08744*	0.00958	0.000	−0.1213	−0.0535
		Golgi	TGB2	0.09875*	0.01250	0.000	0.0545	0.1430
			TGB3	0.08744*	0.00958	0.000	0.0535	0.1213

**The mean difference is significant at the . The highlight emphasizes the numbers that show that Golgi does significantly differ from TGB2 and TGB3 at this significance level*.

### Homogeneous subsets

#### Outer diameter

**Table:**

	Factor	N	Subset for alpha =	
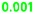
			1	2
Duncan^a,b^	TGB3	21	0.2962	
	TGB2	8	0.2963	
	Golgi	16		0.4775
	Sig.		0.997	1.000

*Means for groups in homogeneous subsets are displayed. The highlight emphasizes the numbers that show that Golgi does significantly differ from TGB2 and TGB3 at this significance level*.

*^a^Uses harmonic mean sample size = 12.759*.

*^b^The group sizes are unequal. The harmonic mean of the group sizes is used. Type I error levels are not guaranteed*.

#### Inner diameter

**Table:**

	Factor	*N*	Subset for alpha =	
			1	2
Duncan^a,b^	TGB2	8	0.1225	
	TGB3	21	0.1338	
	Golgi	16		0.2213
	Sig.		0.328	1.000

*Means for groups in homogeneous subsets are displayed. The highlight emphasizes the numbers that show that Golgi does significantly differ from TGB2 and TGB3 at this significance level*.

*^a^Uses harmonic mean sample size = 12.759*.

*^b^The group sizes are unequal. The harmonic mean of the group sizes is used. Type I error levels are not guaranteed*.

### *Post hoc* tests, *p* < 0.05

**Table:**

Dependent variable		(I) Factor	(J) Factor	Mean difference (I-J)	SE	Sig.	95% Confidence interval	
							Lower bound	Upper bound
Outer diameter	LSD	TGB2	TGB3	0.00006	0.01895	0.998	−0.0382	0.0383
			Golgi	−0.18125*	0.01975	0.000	−0.2211	−0.1414
		TGB3	TGB2	−0.00006	0.01895	0.998	−0.0383	0.0382
			Golgi	−0.18131*	0.01514	0.000	−0.2119	−0.1508
		Golgi	TGB2	0.18125*	0.01975	0.000	0.1414	0.2211
			TGB3	0.18131*	0.01514	0.000	0.1508	0.2119
Inner diameter	LSD	TGB2	TGB3	−0.01131	0.01200	0.351	−0.0355	0.0129
			Golgi	−0.09875*	0.01250	0.000	−0.1240	−0.0735
		TGB3	TGB2	0.01131	0.01200	0.351	−0.0129	0.0355
			Golgi	−0.08744*	0.00958	0.000	−0.1068	−0.0681
		Golgi	TGB2	0.09875*	0.01250	0.000	0.0735	0.1240
			TGB3	0.08744*	0.00958	0.000	0.0681	0.1068

**The mean difference is significant at the . The highlight emphasizes the numbers that show that TGB2 and TGB3 do not differ significantly from each other even at this significance level*.

### Homogeneous subsets

#### Outer diameter

**Table:**

	Factor	*N*	Subset for alpha =	
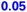
			1	2
Duncan^a,b^	TGB3	21	0.2962	
	TGB2	8	0.2963	
	Golgi	16		0.4775
	Sig.		0.997	1.000

*Means for groups in homogeneous subsets are displayed. The highlight emphasizes the numbers that show that TGB2 and TGB3 do not differ significantly from each other even at this significance level*.

*^a^Uses harmonic mean sample size = 12.759*.

*^b^The group sizes are unequal. The harmonic mean of the group sizes is used. Type I error levels are not guaranteed*.

#### Inner diameter

**Table:**

	Factor	*N*	Subset for alpha =	
			1	2
Duncan^a,b^	TGB2	8	0.1225	
	TGB3	21	0.1338	
	Golgi	16		0.2213
	Sig.		0.328	1.000

*Means for groups in homogeneous subsets are displayed. The highlight emphasizes the numbers that show that TGB2 and TGB3 do not differ significantly from each other even at this significance level*.

*^a^Uses Harmonic Mean Sample Size = 12.759*.

*^b^The group sizes are unequal. The harmonic mean of the group sizes is used. Type I error levels are not guaranteed*.
